# PIK3C2A mRNA functions as a miR-124 sponge to facilitate CD151 expression and enhance malignancy of hepatocellular carcinoma cells

**DOI:** 10.18632/oncotarget.9716

**Published:** 2016-05-30

**Authors:** Tao Liu, Cai-Hua Zu, Shu-Sen Wang, Hong-Li Song, Zheng-Lu Wang, Xin-Nv Xu, Hong-Sheng Liu, Yu-Liang Wang, Zhong-Yang Shen

**Affiliations:** ^1^ Key Laboratory for Critical Care Medicine of the Ministry of Health, Tianjin First Central Hospital, Tianjin, China; ^2^ Organ Transplant Center, Tianjin First Central Hospital, Tianjin, China; ^3^ First Center Clinical College, Tianjin Medical University, Tianjin, China; ^4^ Department of Pathology, Tianjin First Central Hospital, Tianjin, China; ^5^ Biobank of Tianjin First Central Hospital, Tianjin, China; ^6^ Clinical Laboratory, Tianjin First Central Hospital, Tianjin, China

**Keywords:** microRNA, competing endogenous RNA, hepatocellular carcinoma, CD151, PIK3C2A

## Abstract

Competing endogenous RNAs (ceRNAs) are RNA transcripts that can crosstalk with each other by competing for shared microRNAs (miRNAs) through miRNA response elements (MREs). Involved in ceRNA networks, the RNA transcripts may be in a balance, disruption of which could lead to tumorigenesis. Here we reveal a ceRNA interaction between PIK3C2A and CD151 mRNAs in hepatocellular carcinoma (HCC) cells. PIK3C2A is a candidate ceRNA of CD151 because mRNA 3′ untranslated regions (3′UTRs) of these two genes contain miR-124 binding sites. miR-124 is downregulated, while PIK3C2A and CD151 are upregulated in HCC cells compared with normal hepatocytes. Direct and negative regulation of PIK3C2A and CD151 by miR-124 was confirmed in HCC cells. miR-124 and the two potential ceRNAs are all recruited to the RNA-induced silencing complex (RISC). In HCC cell lines QGY- 7703 and SMMC-7721, and normal hepatic cell line HL-7702, miR-124 plays a tumor suppressor role by targeting PIK3C2A and CD151. The MREs within PIK3C2A 3′UTR can independently stimulate CD151 expression level by acting as miR-124 decoys. PIK3C2A MREs enhance HCC cell malignancy by absorbing endogenous miR-124 and activating CD151 in HCC cells. We conclude that PIK3C2A 3′UTR functions as a *trans* activator to stimulate CD151 by competing for miR-124 binding in HCC cells. The collaboration of PIK3C2A and CD151 through ceRNA mechanism may be implicated in HCC initiation and development.

## INTRODUCTION

Hepatocellular carcinoma (HCC) is one of the leading causes of cancer deaths worldwide with over 500,000 new cases being diagnosed each year [1, 2]. The 5-year survival rate is only 20 to 30% in HCC patients after surgical resection mainly due to the aggressiveness, invasiveness and frequent recurrence of HCC [1, 3]. Thus, it is vital to reveal the molecular mechanisms underlying HCC initiation and progression.

As a class of non-coding RNAs (ncRNAs), microRNAs (miRNAs) play important roles in cancer initiation and development [4, 5]. miRNAs are ∼22 nucleotide long, evolutionarily conserved single-stranded RNAs that bind to miRNA response elements (MREs) on target transcripts through sequence complementarity, usually resulting in degradation of the transcript or inhibition of its translation [6]. A single miRNA can regulate thousands of target transcripts, and several miRNAs can corporately bind to separate MREs within a single RNA transcript [6–8]. The RNA transcripts containing same MREs can communicate and regulate each other by competing for a limited pool of miRNAs. These RNAs bearing a same set of MREs are known as competing endogenous RNAs (ceRNAs) [9–12]. Regulation of gene expression through competition for miRNA binding is a general phenomenon [9, 10], and perturbations of ceRNA networks may have profound implications for cancer [13–15]. Until now, the tumor suppressor PTEN has been most extensively validated as competing with a variety of ceRNAs in different cancers. As a pseudogene, PTENP1 performs a biological function by sponging miRNAs from the tumor suppressor PTEN [16]. Following studies in 2011 confirmed VAPA, CNOT6L and ZEB2 as bona fide PTEN ceRNAs [17, 18]. mRNAs are also validated to function as ceRNAs in cancer. For example, HMGA2 promotes lung cancer progression by vying for let-7 binding with TGFBR3, another target of let-7 [19]. Long non-coding RNAs (lncRNAs) also act as ceRNAs to facilitate protein-coding genes [20, 21]. miRNA-mediated gene regulation has been validated in HCC cells [22, 23], and several studies have confirmed the ceRNA mechanism in HCC cells [24, 25]. Thus, we need to consider ceRNAs which add a new layer of complexity to HCC biology. However, the ceRNA mechanism in HCC is still largely unknown.

In this study, we focused on possible ceRNA network in regulation of tetraspanin CD151 expression. We chose CD151 for two reasons. First, as a regulator of laminin-binding integrins, CD151 had been reported to participate in proliferation, epithelial-mesenchymal transition (EMT), migration and invasion of various types of cancer [26] including HCC [27], suggesting its oncogenic potential in HCC progression. Second, according to TargetScanHuman database, a series of MREs were predicted within CD151′s 3′ untranslated region (3′UTR), indicating a probable miRNA-mediated ceRNA network in CD151 regulation. We found that in HCC cells, PIK3C2A 3′UTR acts as a sponge to absorb miR-124 and regulated CD151 expression. The MREs within PIK3C2A 3′UTR are able to promote proliferation and metastasis of HCC cells by regulating CD151 via ceRNA mechanism.

## RESULTS

### CD151, its potential ceRNA PIK3C2A and their possible regulator miR-124 are all dysregulated in HCC cells

**Figure 1:**
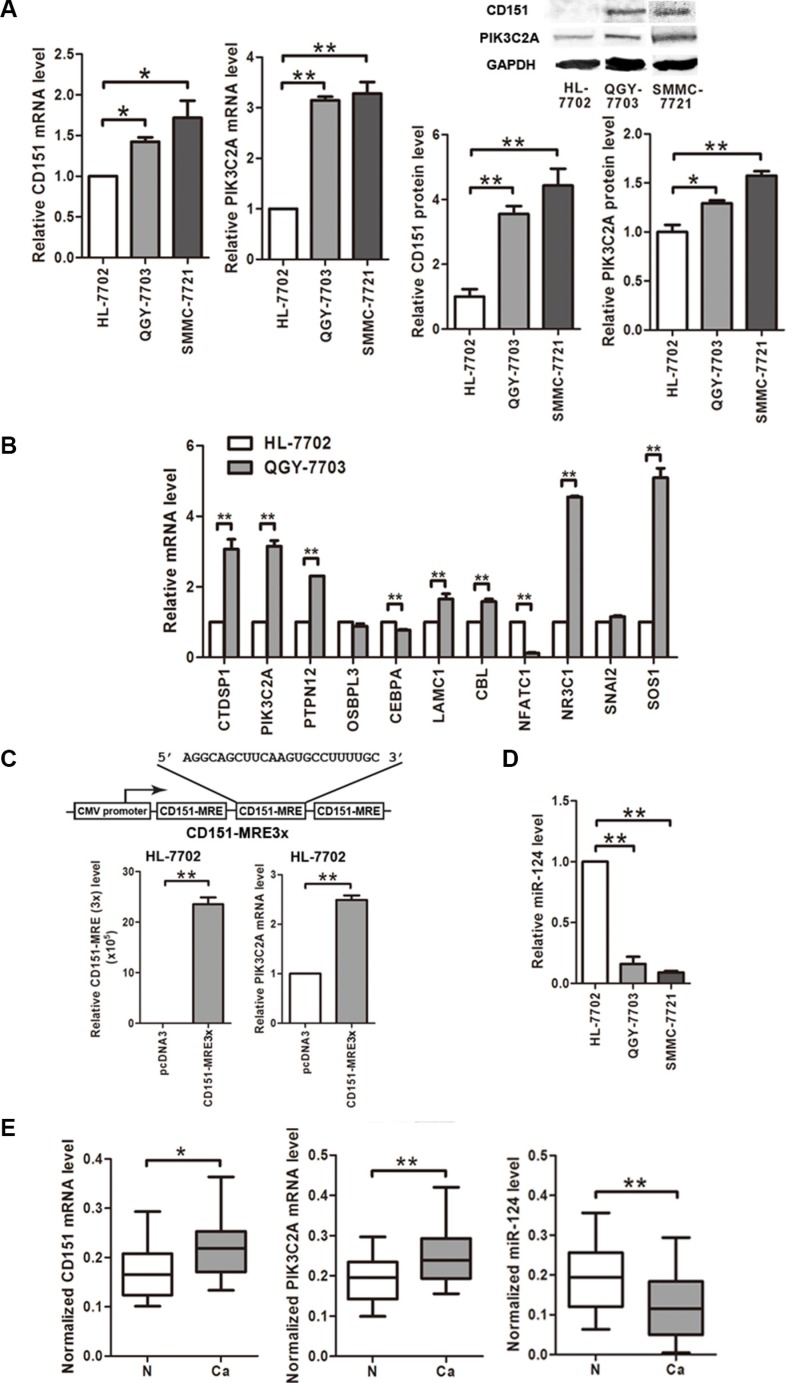
CD151, miR-124 and CD151′s potential ceRNA PIK3C2A are all dysregulated in HCC cells (**A**) mRNA and protein levels of CD151 and PIK3C2A in cell lines were detected by quantitative RT-PCR and Western blot assays, respectively. β-actin mRNA and GAPDH protein were regarded as the endogenous normalizers. (**B**) mRNA levels of 11 CD151′s potential ceRNAs predicted by ceRDB database were detected by quantitative RT-PCR in HL-7702 and QGY-7703 cells. (**C**) A vector expressing the miR-124 reaction element within CD151 3′UTR in triplicate (CD151-MRE3x) was constructed. After transfection, CD151 MREs and PIK3C2A mRNA levels were measured by quantitative RT-PCR. (**D**) miR-124 level in the cell lines was measured by quantitative RT-PCR. U6 snRNA was regarded as the endogenous normalizer. (**E**) CD151 mRNA, PIK3C2A mRNA and miR-124 levels in 20 pairs of HCC tissues (Ca) and normal hepatic tissues (N) were detected by quantitative RT-PCR. (*p < 0.05, **p < 0.01).

At first, we found that CD151 expression level is elevated in the HCC cell lines QGY-7703 and SMMC- 7721 than in the normal hepatic cell line HL- 7702 at both mRNA and protein levels (Figure 1A). We then used ceRDB database to predict the candidate ceRNAs of CD151 and chose 11 cancer-associated genes with higher scores to compare their expression levels between QGY- 7703 and HL-7702 cells. As a result, 7 of the 11 genes were up-regulated and 2 of them were down-regulated in QGY-7703 cells compared with HL- 7702 cells (Figure 1B). Among the up-regulated genes, the oncogene PIK3C2A got a higher score in the list of CD151′s ceRNAs because there are three potential miR- 124 binding sites within 3′UTR of PIK3C2A mRNA. Besides, as a member of PI3K family, PIK3C2A is closely related with signaling pathways involved in HCC progression [28]. So we chose PIK3C2A for further study.

We constructed a vector expressing the sole MRE within CD151 3′UTR (CD151 MRE) in triplicate. Overexpression of triple CD151 MREs led to an enhancement of PIK3C2A mRNA expression in HL-7702 cells (Figure 1C). According to the ceRDB database, CD151 and its potential ceRNA PIK3C2A share miR-124 binding sites. A depressed miR-124 level (Figure 1D) and elevated PIK3C2A mRNA and protein levels (Figure 1A) were confirmed in the two HCC cell lines. Furthermore, the upregulation of CD151 and PIK3C2A mRNAs and the downregulation of miR-124 in HCC cells were also confirmed in the clinical HCC samples and the paired normal hepatic tissues (Figure 1E). These data implicate a possible negative control of CD151 and PIK3C2A expression by miR-124 in HCC cells.

### miR-124 directly targets PIK3C2A and CD151 mRNAs in HCC cells and normal hepatocytes

**Figure 2:**
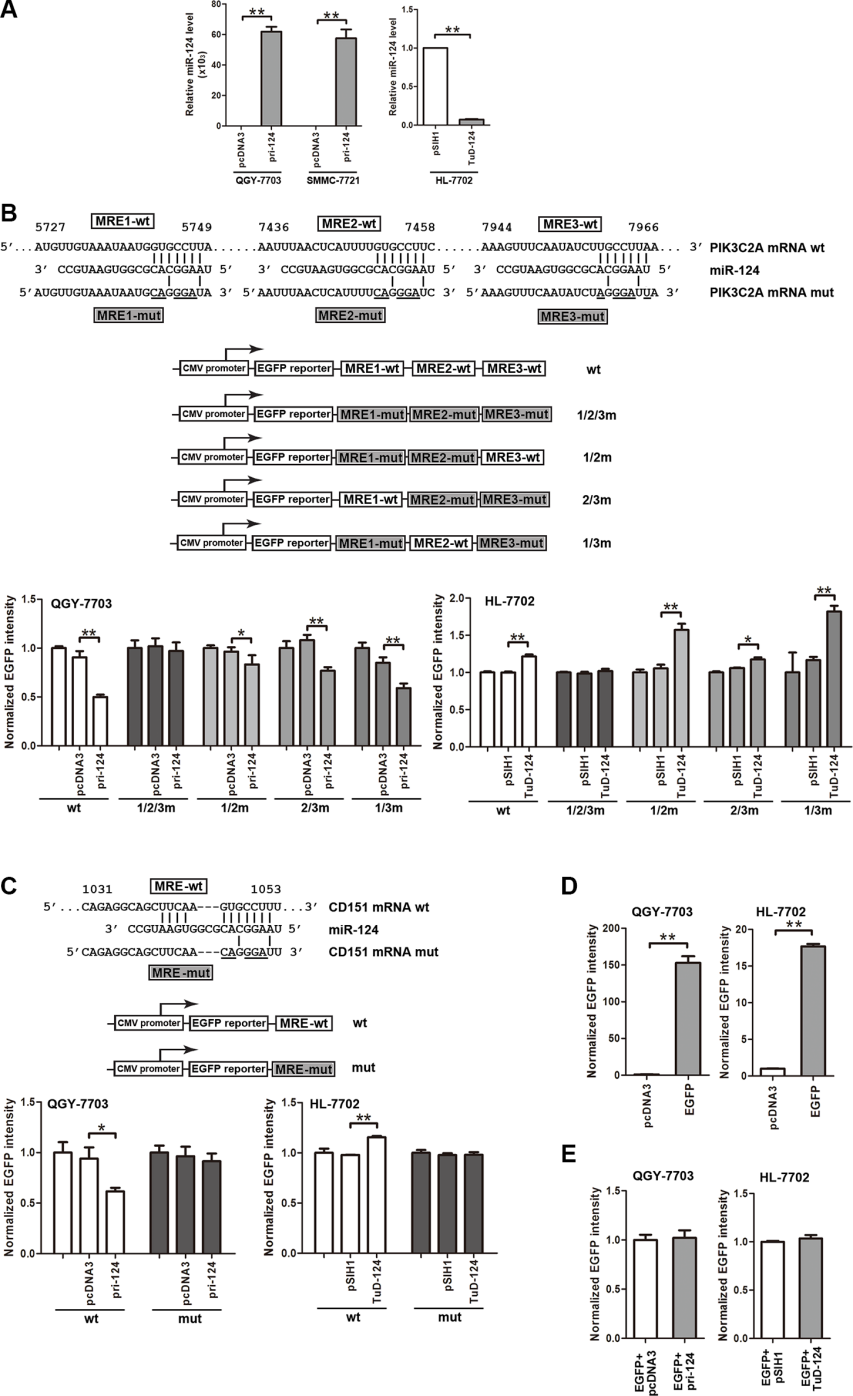
PIK3C2A and CD151 are direct targets of miR-124 (**A**) Primary miR-124 (pri-124) expression vector was used to enhance miR-124 level in QGY-7703 and SMMC-7721 cells, and miR-124 tough decoy (TuD-124) expression vector was used to inhibit miR-124 in HL-7702 cells. (**B**) The wild type (wt) or mutated (mut) PIK3C2A MREs with all the three MREs mutated (1/2/3m) or with every two MREs mutated (1/2m, 2/3m or 1/3m) were cloned at downstream of EGFP coding sequence. The EGFP reporter vector and pri-124 or TuD-124 plasmid were co-transfected into cell lines with RFP expression vector as the normalizer. At 48 h after transfection, cells were lysed and EGFP and RFP activities were detected. The histogram shows normalized EGFP intensity (EGFP/RFP). (**C**) The wild type (wt) or mutated (mut) CD151 MRE was also cloned into pcDNA3/EGFP vector, and EGFP reporter experiments were performed. (**D, E**) To confirm EGFP expression (D) and exclude possible nonspecific effects on EGFP intensity by miR-124 (E), plasmids were transfected into QGY-7703 or HL-7702 cells, and normalized EGFP intensity was measured. (*p < 0.05, **p < 0.01).

The direct targeting on PIK3C2A and CD151 by miR-124 is a precondition for their crosstalk. A miR- 124 ectopic expression vector or a miR-124 “tough decoy” (TuD) [29] was used to enhance or inhibit miR- 124 activity, respectively (Figure 2A). When the three MREs within PIK3C2A 3′UTR (PIK3C2A MREs) were sequentially cloned following the enhanced green fluorescent protein (EGFP) reporter gene, miR-124 attenuated the fluorescence intensity. However, miR-124 aborted to influence EGFP intensity if the three PIK3C2A MREs were all mutated (Figure 2B). we then constructed other three reporter vectors, in each of which two MREs were mutated, leaving the other MRE to be wild type. The EGFP reporter assays showed that each of the three MREs was able to independently bind miR-124 and to negatively control EGFP expression (Figure 2B) revealing that all the three MREs within PIK3C2A mRNA are functional in miR-124-mediated gene silencing. Next, miR-124 also directly bound to the CD151 MRE and inhibited EGFP expression (Figure 2C). In the EGFP reporter assays, effective EGFP expression was confirmed in QGY- 7703 and HL-7702 cells (Figure 2D), and non-specific influence of miR-124 on EGFP expression was excluded (Figure 2E). The above results corroborate suppression of both PIK3C2A and CD151 by miR-124.

**Figure 3:**
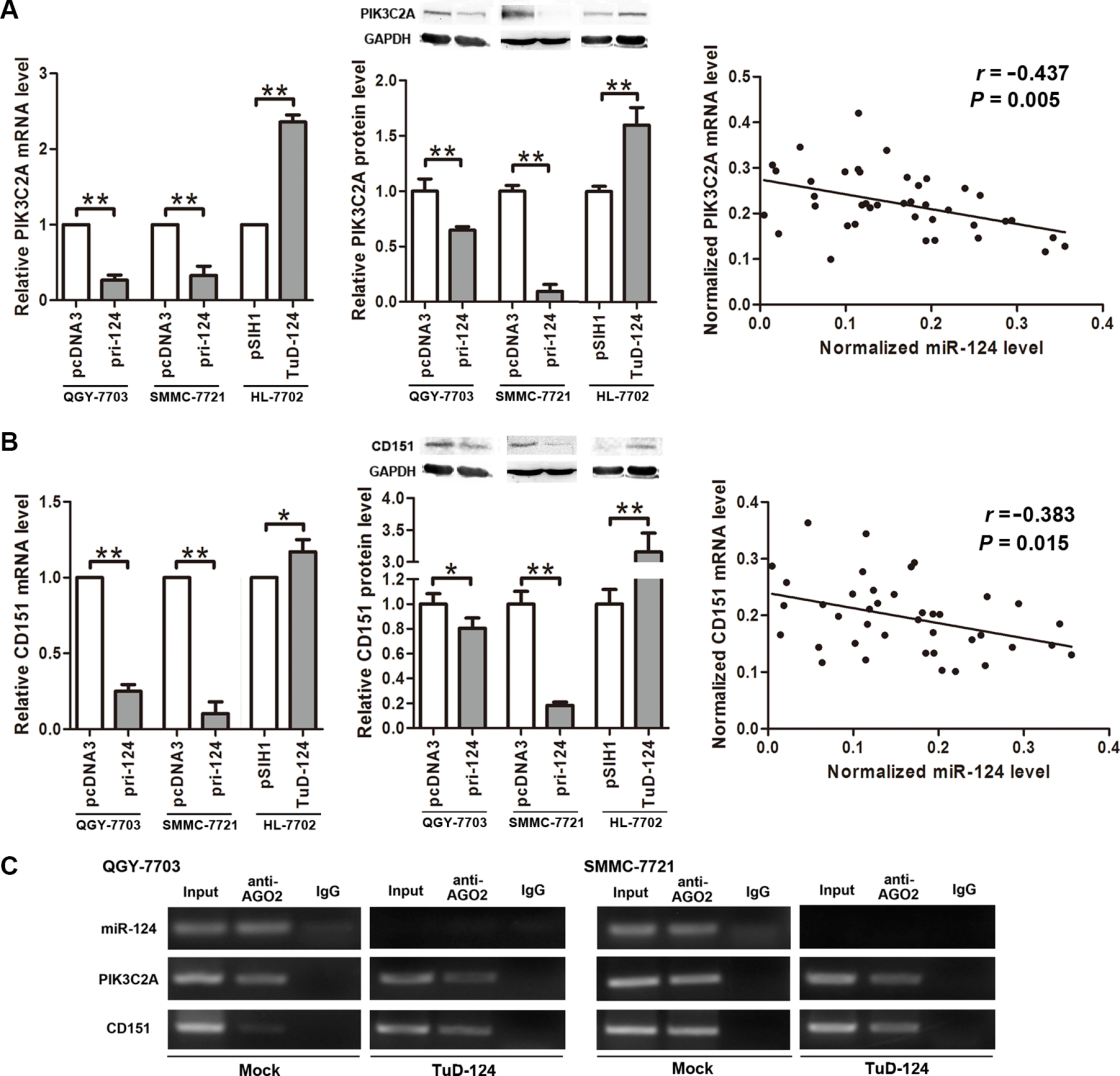
miR-124 negatively regulates PIK3C2A and CD151 expression (**A**) pri-124 or TuD-124 expression vector was transfected into cell lines, and PIK3C2A mRNA and protein levels were detected by quantitative RT-PCR and Western blot assays, respectively. Also, correlation of miR-124 and PIK3C2A mRNA levels in the 20 pairs of clinical tissue samples was analyzed. (**B**) Similarly, the regulation of miR-124 on CD151 expression and linear correlation between miR-124 and CD151 mRNAs were detected and analyzed. (**C**) RIP assay was performed in parental and miR-124-inhibited HCC cell lysate using magnetic beads conjugated AGO2 antibody. miR- 124, PIK3C2A mRNA and CD151 mRNA in the immunoprecipitated RNAs were detected by qRT-PCR. Normal mouse IgG was used as isotype control. (*p < 0.05, **p < 0.01).

Endogenous PIK3C2A expression was inhibited when overexpressing miR-124 in QGY-7703 and SMMC- 7721 cells, and inhibition of miR-124 in HL-7702 cells resulted in PIK3C2A increase on both mRNA and protein levels (Figure 3A). The same regulation pattern was observed on CD151 (Figure 3B). Moreover, in the clinical tissues, miR-124 exhibited negative correlation with PIK3C2A or CD151 mRNA (Figure 3A, 3B). These data demonstrate a negative regulation of endogenous PIK3C2A and CD151 expression by miR-124.

To further validate that the effects on PIK3C2A and CD151 by miR-124 was through miRNA mediated gene silencing, we performed an RNA-binding protein immunoprecipitation (RIP) assay in the parental and miR- 124-inhibited HCC cell lines. The antibody to AGO2 was used to pull-down AGO2 protein, a key member of RNA-induced silencing complex (RISC), and also the AGO2-binding RNAs. As a result, miR-124, PIK3C2A mRNA and CD151 mRNA were all detected in the immunoprecipitated RNAs from the parental HCC cells (Figure 3C), suggesting that miR-124 and its two targets are all recruited into RISC. After miR-124 was inhibited, we did not detect miR-124 in the RNAs pulled-down by anti-AGO2. However, PIK3C2A and CD151 mRNAs were still detectable (Figure 3C), indicating that these two mRNAs may also be controlled by other miRNAs.

### miR-124 suppresses proliferation, migration and invasion of HCC cells via negatively regulating PIK3C2A and CD151

**Figure 4:**
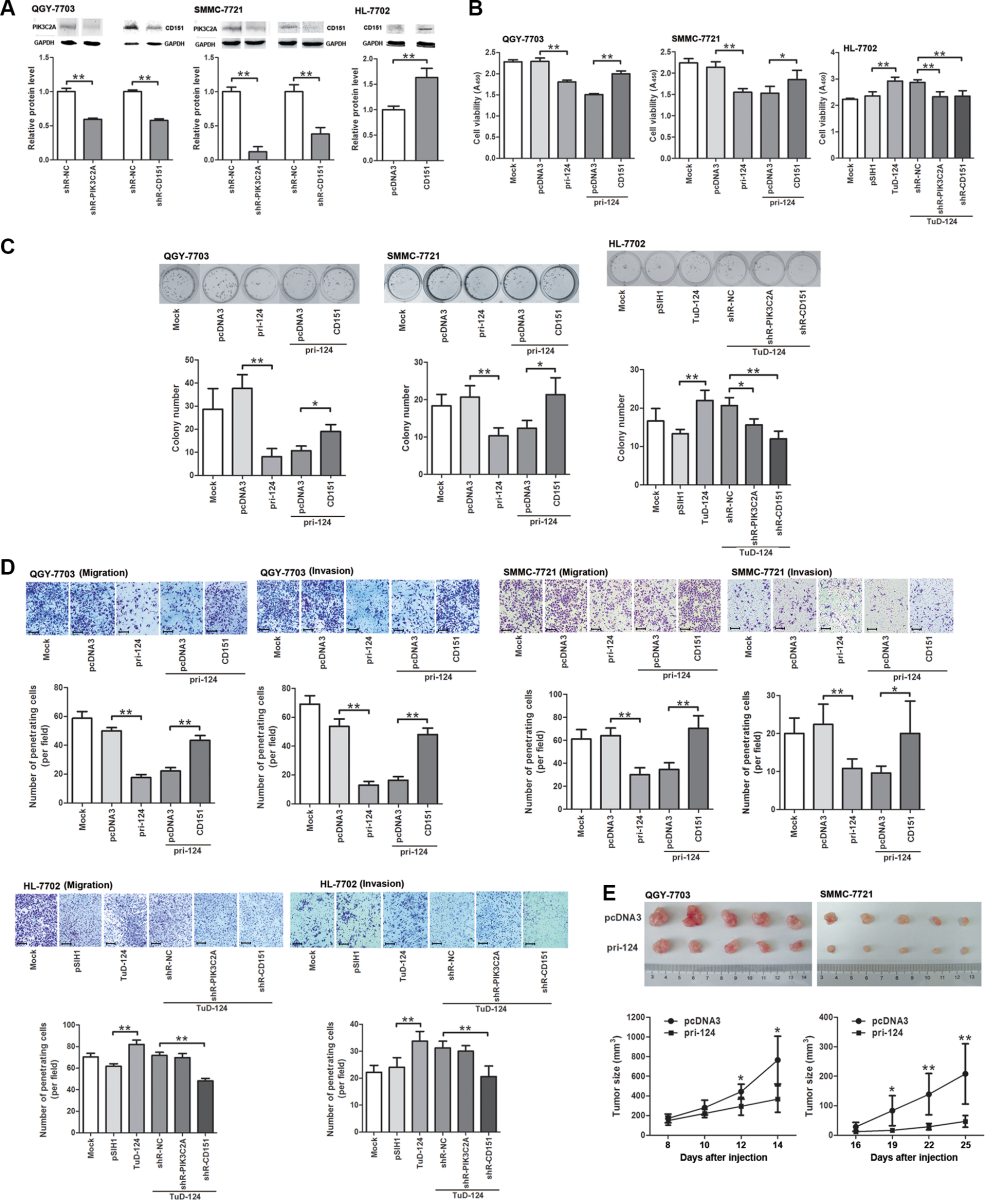
miR-124 alleviates malignancy of HCC cells by regulating PIK3C2A and CD151 (**A**) Endogenous PIK3C2A and CD151 expression was suppressed by shRNA in QGY-7703 and SMMC-7721 cells, and CD151 was forcedly expressed in HL-7702 cells. These alterations were confirmed by Western blot. (**B**) Cell lines were transfected and then seeded in 96-well plates. At 48 h after seeding, CCK-8 assay was performed to evaluate cell viability. The histograms show absorbance of cell cultures at 450 nm (A_450_). (**C**) Cell lines were transfected, counted and seeded in 12-well plates. After 7–8 days (QGY-7703) or 9–10 days (SMMC-7721 or HL-7702), cell colonies were counted and stained using 2% crystal violet. (**D**) Migration and invasion activities of transfected cells were detected using Transwell assays. The penetrating cells in 5 random view fields were counted, and average cell number per field was shown in the histograms (magnification: 100×; the scale bar indicates 100 μm). (**E**) QGY-7703 and SMMC-7721 cells stably expressing miR-124 were injected into right axillary fossa of nude mice to examine growth rate of the xenograft tumors. (Brightness and contrast of the cell images in (C and D) have been modified to make them accordant. *p < 0.05, **p < 0.01).

Given that miR-124 directly targets PIK3C2A and CD151, we next evaluated their influence in HCC cells’ phenotypes. Endogenous PIK3C2A and CD151 were silenced using small hairpin RNAs (shRNAs) in QGY- 7703 and SMMC-7721 cells, and the full-length CDS of CD151 was forcedly expressed in HL-7702 cells (Figure 4A). Increase of miR-124 level attenuated cell viability of the two HCC cell lines, and further expression of CD151 rescued the depressed cell viability caused by miR-124 (Figure 4B). In HL-7702 cells, an enhanced cell viability was observed when miR-124 was inhibited, and the elevated cell activity was restored when either PIK3C2A or CD151 was suppressed (Figure 4B). When detecting colony formation activity of these two cell lines, we obtained similar results (Figure 4C). miR-124 also suppressed migration and invasion of QGY-7703 and HL-7702 cells (Figure 4D). We also found that miR-124 led to downregulation of N-cadherin and upregulation of E-cadherin, suggesting that miR-124 may attenuate EMT of HCC cells (Supplementary Figure S1). Moreover, with a low miR-124 level, CD151 shRNA but not PIK3C2A shRNA could weaken the increased migration and invasion activities caused by miR-124 inhibitor in HL-7702 cells (Figure 4D). *In vivo* studies also suggested that xenograft tumors induced by HCC cells stably expressing miR-124 exhibited a lower growth rate in nude mice (Figure 4E). These experiments illustrated that miR-124 plays a tumor suppressor role in HCC cells by targeting PIK3C2A and CD151.

### PIK3C2A MREs affect CD151 expression through competitively binding miR-124 in HCC cells

**Figure 5:**
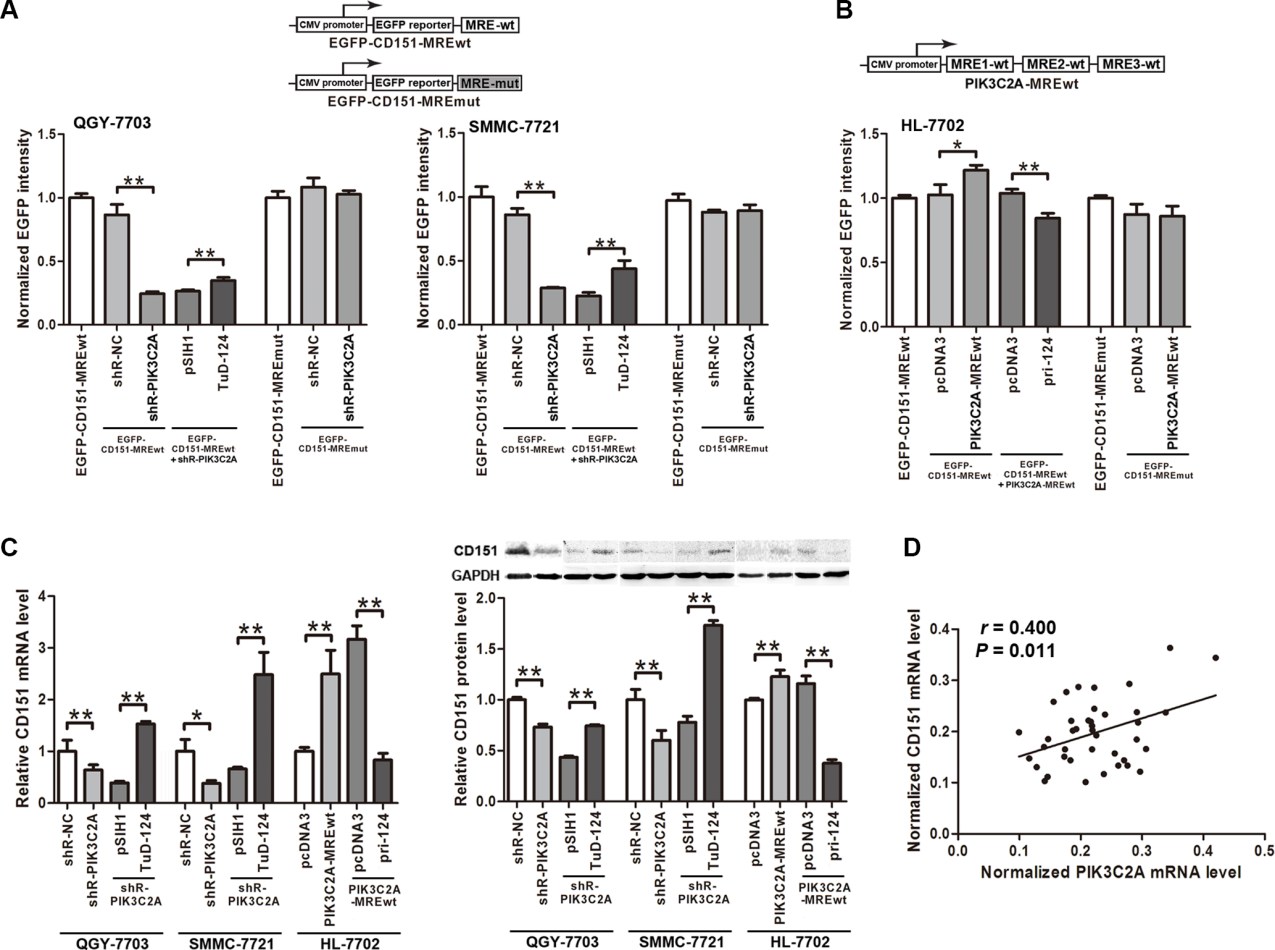
PIK3C2A MREs facilitate CD151 expression (**A**) The pcDNA3/EGFP- CD151-MREwt and -MREmut plasmids were used as reporter vectors, which were co-transfected with other plasmids into QGY-7703 and SMMC-7721 cells to perform fluorescent reporter assays. (**B**) A vector expressing PIK3C2A MREs was constructed, and fluorescent reporter assays were performed in HL-7702 cells to detect the effects of PIK3C2A MREs on CD151 expression. (**C**) PIK3C2A MREs were suppressed followed by suppressing miR-124 in QGY-7703 and SMMC-7721 cells. Also, PIK3C2A MREs were overexpressed followed by overexpressing miR-124 in HL-7702 cells. Endogenous CD151 in these cells was measured on both mRNA and protein levels. (**D**) PIK3C2A mRNA and CD151 mRNA levels in clinical tissues were detected, and linear correlation between these two RNA transcripts was analyzed. (*p < 0.05, **p < 0.01).

We then used the EGFP-CD151-MRE reporter vectors to detect the effects of PIK3C2A MRE on CD151 expression. EGFP intensity obviously decreased when PIK3C2A shRNA was transfected into QGY- 7703 and SMMC-7721 cells to degrade PIK3C2A mRNA. Importantly, further expression of miR-124 partly saved the depressed EGFP level. When the CD151 MRE sequence within the reporter vector was mutated, shR- PIK3C2A could no longer affect EGFP intensity (Figure 5A). Similar experiments in HL-7702 further verified that the three PIK3C2A MREs could independently stimulate CD151 expression, in which miR- 124 was also involved (Figure 5B).

We then detected influence of PIK3C2A MREs on endogenous CD151 expression. Inhibition of PIK3C2A mRNA led to a CD151 level decrease in QGY-7703 and SMMC-7721 cells, which was further reversed by miR- 124 suppression. On the other hand, ectopic expression of PIK3C2A MREs in HL-7702 cells caused an elevated CD151 level, and subsequent miR-124 expression restored it (Figure 5C). A linear positive correlation between PIK3C2A and CD151 mRNAs in the 20 pairs of HCC and normal hepatic tissues were also confirmed (Figure 5D). Furthermore, their positive correlation also exists in other two microarray-based studies containing large number of HCC and non-tumor hepatic tissues (GEO datasets GDS4887 and GSE36376; Supplementary Table S1 and Supplementary Figure S2) [30]. The above data support the hypothesis that PIK3C2A MREs are enough to competitively absorb miR-124 and to up- regulate CD151 expression.

### PIK3C2A MREs enhance HCC cell malignancy through absorbing miR-124 and subsequent upregulation of CD151

**Figure 6:**
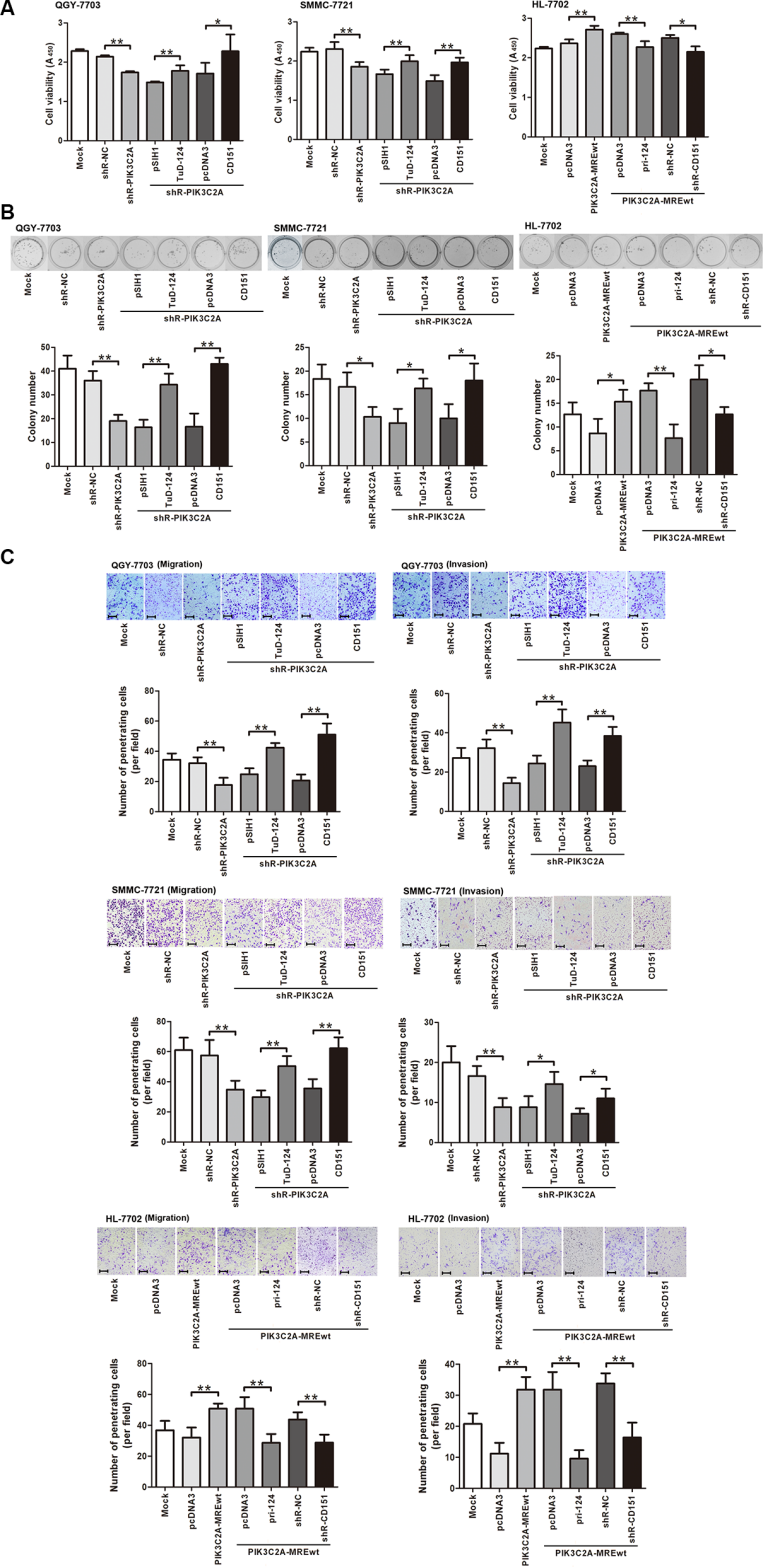
PIK3C2A MREs enhance HCC cell malignancy by alleviating miR-124 mediated CD151 suppression (**A**) Endogenous PIK3C2A MREs were inhibited by shRNA (shR-PIK3C2A), and miR-124 repressor (TuD-124) or CD151 ectopic expression vector was further transfected in QGY-7703 and SMMC-7721 cells. In HL-7702 cells, PIK3C2A MREs were overexpressed and pri-124 or CD151 shRNA expression vector (shR-CD151) was further transfected. CCK-8 assay was performed to evaluate cell viability. (**B**) Cell lines were transfected as described in (A), and colony formation activity was detected. (**C**) Cell lines were transfected as described in (A), and migration and invasion activities of these cells were measured by Transwell assays (magnification: 100×; the scale bar indicates 100 μm). (Brightness and contrast of the cell images in B and C have been modified to make them accordant. *p < 0.05, **p < 0.01).

After validating the regulation of PIK3C2A MREs on CD151, we then evaluated the role of PIK3C2A MREs in HCC malignancy. shRNA mediated PIK3C2A mRNA degradation resulted in a decreased viability of QGY-7703 and SMMC-7721 cells, and this impact could be reversed sequentially by either inhibiting miR-124 or overexpressing CD151. In HL-7702 cells, special expression of PIK3C2A MREs enhanced cell viability, which was restored by expression of miR-124 or suppression of CD151 (Figure 6A). Similar phenomena were observed in colony formation assays (Figure 6B). Furthermore, transwell experiments suggested that PIK3C2A MREs was able to positively regulate migration and invasion activities, and these effects could also be reversed by artificially altering miR-124 or CD151 levels (Figure 6C). These results elucidated that PIK3C2A mRNA acts as a miR-124 decoy to regulate CD151 and to affect HCC malignant phenotypes.

## DISCUSSION

Aberrant ceRNA networks have been linked to tumorigenesis [13–15]. In this study, we revealed a miR-124 mediated crosstalk between PIK3C2A and CD151 mRNAs in HCC. To validate the ceRNA network, it was principal to confirm that both the two RNA transcripts could bind endogenous miR-124. First, PIK3C2A was predicted to be a candidate ceRNA of CD151 because their mRNA transcripts bear miR-124 binding sites according to bioinformatic database. Nevertheless, experimental evidence was needed to validate PIK3C2A as a CD151′s bona fide ceRNA. Second, fluorescent reporter assays determined the direct interaction between miR-124 and the two mRNAs. Third, miR-124 and its two targets were all dysregulated in HCC cells. In cell lines and HCC clinical tissues, PIK3C2A and CD151 exhibited negative correlation with miR-124 level, supporting an actual regulation of the two mRNAs by miR-124. Fourth, miR-124 and the two candidate ceRNAs were symultaneously recruited into AGO2 within RISC, a key locus of miRNA mediated target RNA degradation or translational repression [6, 31]. These facts provide essential conditions for ceRNA crosstalk between PIK3C2A and CD151.

ceRNA interactions can be either symmetrical, whereby two ceRNAs co-regulate each other, or asymmetrical, whereby one ceRNA regulates expression of the other but not the reverse [32]. We found that the crosstalk between PIK3C2A and CD151 in HCC cells was asymmetrical. Alteration of PIK3C2A MREs led to a coordinate CD151 expression, and change of CD151 MRE, however, did not result in any detectable variation of PIK3C2A level (data not shown). PIK3C2A mRNA bears as many as three MREs within its 3′UTR, and each MRE was responsible for miR-124 binding and PIK3C2A suppression (Figure 2B). Thus, we presumed that the MRE number is a factor that may influence effectiveness of ceRNA [10]. With three MREs, PIK3C2A 3′UTR can absorb large abundance of miR-124 molecules and tend to affect CD151. However, CD151 3′UTR has little impact on PIK3C2A expression possibly due to low titrimetric ability of the single MRE within it. This hypothesis was also confirmed by our experiment, in which PIK3C2A level was also responsive to change of CD151 MRE if the MRE sequence was cloned into the vector in triplicate (Figure 1C).

In the two HCC cell lines, we used shR-PIK3C2A to reduce endogenous PIK3C2A MRE level by reason that double-stranded siRNAs can guide cleavage of target RNA [33]. shR-PIK3C2A led to degradation of PIK3C2A MREs and more miR-124 was, as a result, released to bind CD151 MRE and suppress CD151 expression. To confirm this, we found that miR-124 inhibitor can partly rescue CD151 level from shR-PIK3C2A mediated depression. Here we should note that besides PIK3C2A MREs degradation, shRNA also suppresses PIK3C2A protein expression. We should exclude the possible that suppression of CD151 was due to the silence of PIK3C2A protein expression. This was performed in HL-7702 cells, in which only PIK3C2A MREs were overexpressed. The elevated PIK3C2A MREs rescued CD151 from miR-124 induced suppression, because forced miR-124 expression could again reduce CD151 level. From these data, we presume that PIK3C2A MREs affect CD151 level by competing for miR-124 binding.

**Figure 7:**
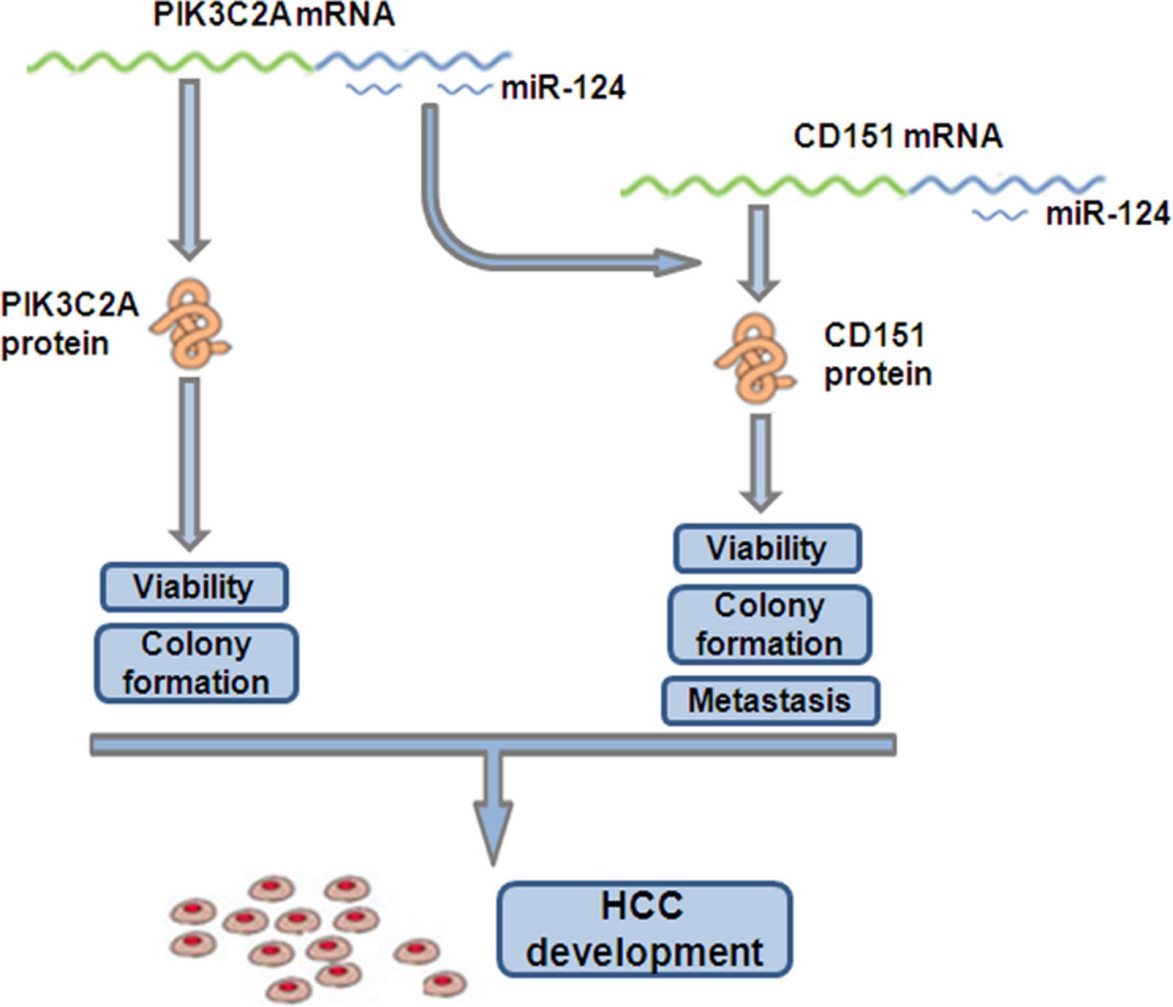
ceRNA crosstalk between PIK3C2A and CD151 in HCC cells As ceRNAs, PIK3C2A and CD151 mRNAs competitively bind miR-124 pool in HCC cells. PIK3C2A mRNA encodes protein to facilitate HCC proliferation. Moreover, PIK3C2A 3′UTR can absorb miR-124 and alleviate miR-124 mediated CD151 suppression. The raised CD151 protein expression, as a result, further promotes HCC proliferation and metastasis.

CD151 belongs to an evolutionarily conserved transmembrane-4 family known as tetraspanins [34] and is principally involved in all types of integrin-mediated cellular behavior [35]. As an oncogene, CD151 not only supports growth of various types of tumors but also regulates tumor cell spreading, migration and invasion [26, 36–38] and facilitates metastatic process [39]. Our data demonstrated an oncogenic role of CD151 in HCC cells, in which CD151 enhances proliferation, migration and invasion. In mammary cancer cells, the CD151-α3β1 integrin complex participates in invasive migration, which is blocked by PI3K inhibitor, suggesting the involvement of tetraspanin-integrin complex in cell invasion via PI3K-dependent pathway [40]. Another research also demonstrated the role of tetraspanin in regulation of the integrin-dependent PI3K signaling pathway [41]. In this study, we illustrated a ceRNA interaction mechanism between PIK3C2A, a member of PI3K family, and CD151. In HCC cells, the three miR-124 binding sites within PIK3C2A act as a sponge to absorb miR-124, liberating CD151 from miR-124 targeting. The non-coding PIK3C2A MREs contributes, at least in part, in this process, because in HL-7702 cells, overexpression just the PIK3C2A MREs was able to enhance cell malignancy, which was also miR-124 or CD151 dependent (Figure 6). Interestingly, with a low miR-124 level, degradation of PIK3C2A mRNA suppressed cell proliferation (Figure 4B, 4C) but did not affect migration and invasion (Figure 4D). We presumed that PIK3C2A promotes HCC cell proliferation but not metastasis via its protein-coding function [28], while its non-coding region enhances both proliferation and metastasis of HCC cells by facilitating CD151 expression (Figure 7). The function of PIK3C2A coding region was not measured because we were not able to successfully amplify the 5061-bp full-length PIK3C2A coding region. Alternatively, the exact role of PIK3C2A MREs was mainly measured by its ectopic expression vector in HL-7702 cells.

RNA transcripts which serve as endogenous miRNA sponges contain pseudogenes, long non-coding RNAs (lncRNAs), circular RNAs (circRNAs) and the UTR of mRNAs [10, 13]. This study suggested a ceRNA crosstalk between two mRNA transcripts. PIK3C2A 3′UTR acts as a *trans* modulator of CD151 expression through miR-124 binding, conferring an additional non-protein-coding role to protein-coding PIK3C2A mRNA. It should be mentioned that one shortcoming of the ceRNA prediction database ceRDB is that it considers only mRNAs with shared MREs for the ceRNA network [42]. Many ncRNAs may also act as CD151′s functional ceRNAs, which should be elucidated in the following studies.

Collectively, this study revealed a miR- 124- dependent ceRNA crosstalk between PIK3C2A and CD151 mRNAs. Upregulated PIK3C2A expression (i.e. transcriptional activation) results in a redistribution of miR-124 and facilitates CD151 expression in hepatocytes (Figure 7). The coordinate expression of these two genes may allow us to better understand the mechanism of HCC initiation and development.

## MATERIALS AND METHODS

### Cell lines, transfection and clinical tissue samples

Human HCC cell lines QGY-7703, SMMC-7721 and normal hepatic cell line HL-7702 were obtained from the Cell Bank of Saierbio Inc. (Tianjin, China), and these cell lines were originally obtained from the Cell Bank of Shanghai Institutes for Biological Sciences, Chinese Academy of Sciences (Shanghai, China). At 2 weeks before passaged in our laboratory, both the cell lines were characterized using vitality test and mycoplasma detection by Saierbio Inc. The cell lines were maintained in RPMI- 1640 medium (Solarbio, Beijing, China) supplemented with 10% (QGY-7703 and SMMC-7721) or 15% (HL- 7702) fetal bovine serum (FBS, Gibco, Carlsbad, CA, USA) at 37°C in a humidified chamber supplemented with 5% CO_2_. Transfection of plasmids was performed with Lipofectamine 2000 Reagent (Invitrogen, Carlsbad, CA, USA) according to the manufacturer's instruction.

Twenty pairs of human HCC tissues and normal hepatic tissues, confirmed by pathological analysis, were obtained from the Biobank of Tianjin First Center Hospital with the patients' informed consent. This study was approved by the Ethics Committee of Tianjin First Central Hospital. The normal hepatic tissues were the distal end of the operative excision far away from the tumor. The tissue samples were snap-frozen in liquid nitrogen immediately after surgical resection and then stored at −80°C until use.

### Bioinformatics

ceRDB database [42] (http://www.oncomir.umn.edu/cefinder/basic_search.php) was used to predict CD151′s potential ceRNAs. The miRNA binding sequences within the target mRNAs were searched by TargetScanHuman Release 7.0 [43].

### Vector construction

To construct the pcDNA3/CD151-MRE3x vector expressing CD151 MRE in triplicate, a double-strand DNA (dsDNA) fragment was obtained by annealing reaction of CD151-MRE3x-top and -bottom. The DNA fragment was cloned into a pcDNA3.1(+) vector (Invitrogen) at the *Bam*HI and *Eco*RI sites (All the endonucleases and T4 DNA ligase used in this study were purchased from TaKaRa, Otsu, Shiga, Japan).

To construct the miR-124 ectopic expression vector pcDNA3/pri-124, a 190-bp dsDNA fragment containing miR-124 precursor was amplified by PCR using human genomic DNA as template and using pri-124-S and -A as primers. The amplified fragment was cloned into a pcDNA3.1(+) vector at the *Bam*HI and *Eco*RI sites.

The miR-124 TuD [29] was used to suppress miR-124 activity. The TuD-124 expression vector pSIH1-H1-puro/TuD-124 was constructed by directly synthesizing a 134-bp TuD-124 sequence into a pcDNA3.1(+) vector at the *Bam*HI and *Eco*RI sites.

To construct the EGFP reporter vectors, we first amplified the 720-bp whole EGFP CDS by PCR using pEGFP-N1 plasmid (Clontech, Otsu, Shiga, Japan) as template and using EGFP-CDS-S and -A as primers. The amplified fragment was cloned into a pcDNA3.1(+) vector at the *Hind*III and *Bam*HI sites to generate pcDNA3/EGFP vector. Then the wild type or mutated MREs (PIK3C2A MREs 1/2/3 and CD151 MREs) were firstly obtained by annealing reactions of top and bottom oligonucleotides and then cloned into the pcDNA3/EGFP vector at the endonuclease sites (*Bam*HI, *Eco*RI, *Xho*I and *Xba*I) downstream of EGFP CDS. Also, the PIK3C2A wild type MREs expression vector pcDNA3/PIK3C2A-MREwt was constructed by cloning the annealed PIK3C2A MREs into a pcDNA3.1(+) vector without EGFP CDS.

To construct shRNA expression vectors, three ∼65 bp dsDNA fragments (shR-PIK3C2A, shR-CD151 and a control shR-NC) were obtained via annealing reactions. The fragments were then respectively cloned into a pSilencer 2.1 neo vector (Ambion, Austin, TX, USA) at the *Bam*HI and *Hind*III sites.

To construct the CD151 ectopic expression vector, the full-length CD151 CDS was amplified by PCR using the oligo dT primed cDNA from QGY-7703 cells as template and CD151-CDS-S and -A as primers. The amplified fragment was cloned into a pcDNA3.1(+) vector at the *Eco*RI and *Xba*I sites.

All the primers and oligonucleotides were ordered from AuGCT Inc. (Beijing, China) and sequence of these DNAs is provided in Supplementary Table S2. All the constructed plasmids were validated using DNA sequencing by AuGCT Inc.

### Extraction of RNA and protein

The large (> 200 nt) and small (< 200 nt) RNAs were extracted and isolated using *mir*Vana^TM^ miRNA Isolation Kit (Ambion) according to the manufacturer's instructions. To extract protein from cell lines, the cells were lysed using RIPA lysis buffer (150 mM NaCl, 50 mM Tris-HCl pH 7.2, 1% Triton X-100 and 0.1% SDS). After centrifugation, the undissolved cell components were removed and the cellular total proteins were obtained.

### Quantitative RT-PCR (qRT-PCR)

Quantitation of miR-124 and an endogenous control U6 snRNA was performed using a stem-loop RT- PCR assay [44]. Briefly, 2 μg small RNA was reverse transcribed into cDNA using a PrimeScript II 1st Strand cDNA Synthesis Kit (TaKaRa) with miR-124-RT or U6- RT primers. The cDNAs were then used for miR-124 and U6 snRNA quantification by PCR.

For mRNA quantification of protein-coding genes and the triple CD151 MREs, 5 μg large RNA was reverse transcribed into cDNA using oligo dT primers. The cDNAs were then used for amplification of target RNAs and an endogenous control β-actin.

All the quantitative PCR reactions were performed using the SYBR *Premix Ex Taq* II (TaKaRa) on a 7300 Real Time PCR System (Applied Biosystems, Grand Island, NY, USA). Gene expression was analysed using the ΔΔCt method.

### Western blot assay

For detecting target protein levels, the cellular total proteins were resolved on an SDS denaturing polyacrylamide gel and then transferred onto a nitrocellulose membrane. Antibodies to CD151 (Santa Cruz, Dallas, TX, USA, Cat# sc-271216), PIK3C2A (Santa Cruz, Cat# sc-365290) or an endogenous control GAPDH (Signalway Antibody, College Park, MD, USA) were incubated with the membranes overnight at 4°C. The membranes were then washed and incubated with horseradish peroxidase (HRP)-conjugated secondary antibodies (Goat anti-mouse IgG, Genomapping, Tianjin, China). Protein expression was assessed by enhanced chemiluminescence and the bands were captured by a FluorChem FC2 Imaging System (Alpha Innotech, Kasendorf, Germany). The band intensity was analyzed by an AlphaView SA V3.4.0 (ProteinSimple, San Jose, CA, USA).

### Fluorescent reporter assays

Cells were transfected with an EGFP reporter plasmid alone or with other plasmids (miR-124 expression or suppression vector, PIK3C2A wild type MREs expression vector, etc.). In each group, identical amounts of red fluorescent protein (RFP) expression vector pDsRed2-N1 (Clontech) were co-transfected to serve as the loading control. After 48 h, the cells were lysed with RIPA lysis buffer and fluorescent intensity was measured using an EnSpire^TM^ Multilabel Reader (PerkinElmer, Waltham, MA, USA).

### RIP assay

RIP assay was performed using MagnaRIP^TM^ RNA-Binding Protein Immunoprecipitation Kit (Millipore, Billerica, MA, USA) following the manufacturer's instruction. miR-124, PIK3C2A mRNA and CD151 mRNA in the immunoprecipitated RNAs were then analyzed via quantitative RT-PCR.

### Cell counting kit-8 (CCK-8) cell viability assay

Transfected cells were seeded in a 96-well plate. At 48 h after seeding, CCK-8 (Dojindo, Kumamoto, Japan) was added to the cells and absorbance of the cell culture at 450 nm was measured using an EnSpire^TM^ Multilabel Reader (PerkinElmer).

### Colony formation assay

To detect the cell colony formation activity, 100 (QGY-7703), 120 (SMMC-7721) or 200 (HL-7702) cells were seeded into each well of a 12-well plate. After 7–8 days (QGY-7703) or 9–10 days (SMMC-7721 and HL- 7702), cell colonies were counted if a colony contained more than 50 cells. The colonies were finally stained using 2% crystal violet.

### Transwell migration and invasion assays

Transwells (Corning, Corning, NY, USA, Cat#3422) with Matrigel (BD, Franklin Lakes, NJ, USA, for invasion assays) or without Matrigel (for Migration assays) were inserted into 24-well plate. An amount of 2 × 10^4^ (for QGY- 7703 and SMMC-7721 migration assays), 4 × 10^4^ (for QGY-7703 and SMMC-7721 invasion assays or HL- 7702 migration assay) or 8 × 10^4^ (for HL-7702 invasion assay) cells within 200 μL serum-free medium were added to the upper chamber of the well, and the lower chamber was filled with complete medium. The cells were allowed to penetrate at 37°C, 5% CO_2_ for 12 h (QGY-7703) or 24 h (SMMC- 7721 and HL-7702). Then, the penetrated cells attached to the lower surface were stained with 2% crystal violet and were observed under a Nikon Ni-U microscope. Cells in 5 random view fields at 100× magnification were counted.

### *In vivo* xenograft tumor studies

QGY-7703 and SMMC-7721 cells were transfected with pcDNA3/pri-124 or the control vector pcDNA3, followed by selection for 20 (QGY-7703) or 28 (SMMC- 7721) days in complete RPMI-1640 medium supplemented with 300 μg/mL Geneticin (Gibco) to establish HCC cell lines stably overexpressing miR- 124. Overexpression of miR-124 in the selected cells were confirmed by qRT-PCR. The cells (6 × 10^6^ in 200 μL) were inoculated into right axillary fossa of male athymic BALB/c nude mice aged 4–5 weeks. Tumor size was monitored by measuring length and width with calipers, and tumor volume was calculated with the formula: (L×W^2^) × 0.5, in which L is the length and W is the width of tumor. At the 14th (QGY-7703) or 25th (SMMC-7721) day after injection, the mice were sacrificed and the tumors were isolated. The mice used in this experiment were handled in accordance with NIH Animal Care and Use Committe Regulations, and this study was approved by the Ethics Committee of Tianjin First Central Hospital.

### Statistical analysis

All the data were reported as mean ± standard deviation (SD) collected from three independent experiments with three technical replicates (five in CCK- 8 assays) in each experiment. The hypothesis test for significance between two groups utilized Student's *t* test; for three or more groups, a one-way analysis of variance (ANOVA) was used, followed by Student-Newman-Keuls *q* test for comparing each two groups. The statistical significance was set at *p* ≤ 0.05. Figure drawing and data processing were performed using GraphPad Prism v5.0 (GraphPad Software, La Jolla, CA, USA).

## SUPPLEMENTARY MATERIALS FIGURES AND TABLES





## References

[1] El-Serag HB (2011). Hepatocellular carcinoma. N Engl J Med.

[2] Siegel R, Ma J, Zou Z, Jemal A (2014). Cancer statistics, 2014. CA Cancer J Clin.

[3] Pang RW, Joh JW, Johnson PJ, Monden M, Pawlik TM, Poon RT (2008). Biology of hepatocellular carcinoma. Ann Surg Oncol.

[4] Farazi TA, Spitzer JI, Morozov P, Tuschl T (2011). miRNAs in human cancer. J Pathol.

[5] Lujambio A, Lowe SW (2012). The microcosmos of cancer. Nature.

[6] Bartel DP (2009). MicroRNAs: target recognition and regulatory functions. Cell.

[7] Friedman RC, Farh KK, Burge CB, Bartel DP (2009). Most mammalian mRNAs are conserved targets of microRNAs. Genome Res.

[8] Ebert MS, Sharp PA (2012). Roles for microRNAs in conferring robustness to biological processes. Cell.

[9] Salmena L, Poliseno L, Tay Y, Kats L, Pandolfi PP (2011). A ceRNA hypothesis: the Rosetta Stone of a hidden RNA language?. Cell.

[10] Tay Y, Rinn J, Pandolfi PP (2014). The multilayered complexity of ceRNA crosstalk and competition. Nature.

[11] Sen R, Ghosal S, Das S, Balti S, Chakrabarti J (2014). Competing endogenous RNA: the key to posttranscriptional regulation. ScientificWorldJournal.

[12] Kartha RV, Subramanian S (2014). Competing endogenous RNAs (ceRNAs): new entrants to the intricacies of gene regulation. Front Genet.

[13] Su X, Xing J, Wang Z, Chen L, Cui M, Jiang B (2013). microRNAs and ceRNAs: RNA networks in pathogenesis of cancer. Chin J Cancer Res.

[14] de Giorgio A, Krell J, Harding V, Stebbing J, Castellano L (2013). Emerging roles of competing endogenous RNAs in cancer: insights from the regulation of PTEN. Mol Cell Biol.

[15] Sanchez-Mejias A, Tay Y (2015). Competing endogenous RNA networks: tying the essential knots for cancer biology and therapeutics. J Hematol Oncol.

[16] Poliseno L, Salmena L, Zhang J, Carver B, Haveman WJ, Pandolfi PP (2010). A coding-independent function of gene and pseudogene mRNAs regulates tumour biology. Nature.

[17] Tay Y, Kats L, Salmena L, Weiss D, Tan SM, Ala U, Karreth F, Poliseno L, Provero P, Di Cunto F, Lieberman J, Rigoutsos I, Pandolfi PP (2011). Coding-independent regulation of the tumor suppressor PTEN by competing endogenous mRNAs. Cell.

[18] Karreth FA, Tay Y, Perna D, Ala U, Tan SM, Rust AG, DeNicola G, Webster KA, Weiss D, Perez-Mancera PA, Krauthammer M, Halaban R, Provero P (2011). *In vivo* identification of tumor- suppressive PTEN ceRNAs in an oncogenic BRAF-induced mouse model of melanoma. Cell.

[19] Kumar MS, Armenteros-Monterroso E, East P, Chakravorty P, Matthews N, Winslow MM, Downward J (2014). HMGA2 functions as a competing endogenous RNA to promote lung cancer progression. Nature.

[20] Liu XH, Sun M, Nie FQ, Ge YB, Zhang EB, Yin DD, Kong R, Xia R, Lu KH, Li JH, De W, Wang KM, Wang ZX (2014). Lnc RNA HOTAIR functions as a competing endogenous RNA to regulate HER2 expression by sponging miR-331-3p in gastric cancer. Mol Cancer.

[21] Ma MZ, Chu BF, Zhang Y, Weng MZ, Qin YY, Gong W, Quan ZW (2015). Long non-coding RNA CCAT1 promotes gallbladder cancer development via negative modulation of miRNA-218-5p. Cell Death Dis.

[22] D'Anzeo M, Faloppi L, Scartozzi M, Giampieri R, Bianconi M, Del Prete M, Silvestris N, Cascinu S (2014). The role of micro-RNAs in hepatocellular carcinoma: from molecular biology to treatment. Molecules.

[23] Callegari E, Elamin BK, Sabbioni S, Gramantieri L, Negrini M (2013). Role of microRNAs in hepatocellular carcinoma: a clinical perspective. Onco Targets Ther.

[24] Tang J, Zhuo H, Zhang X, Jiang R, Ji J, Deng L, Qian X, Zhang F, Sun B (2014). A novel biomarker Linc00974 interacting with KRT19 promotes proliferation and metastasis in hepatocellular carcinoma. Cell Death Dis.

[25] Peng H, Ishida M, Li L, Saito A, Kamiya A, Hamilton JP, Fu R, Olaru AV, An F, Popescu I, Iacob R, Dima S, Alexandrescu ST (2015). Pseudogene INTS6P1 regulates its cognate gene INTS6 through competitive binding of miR-17-5p in hepatocellular carcinoma. Oncotarget.

[26] Sadej R, Grudowska A, Turczyk L, Kordek R, Romanska HM (2014). CD151 in cancer progression and metastasis: a complex scenario. Lab Invest.

[27] Ke AW, Shi GM, Zhou J, Huang XY, Shi YH, Ding ZB, Wang XY, Devbhandari RP, Fan J (2011). CD151 amplifies signaling by integrin alpha6beta1 to PI3K and induces the epithelial-mesenchymal transition in HCC cells. Gastroenterology.

[28] Ng SK, Neo SY, Yap YW, Karuturi RK, Loh ES, Liau KH, Ren EC (2009). Ablation of phosphoinositide-3-kinase class II alpha suppresses hepatoma cell proliferation. Biochem Biophys Res Commun.

[29] Haraguchi T, Ozaki Y, Iba H (2009). Vectors expressing efficient RNA decoys achieve the long-term suppression of specific microRNA activity in mammalian cells. Nucleic Acids Res.

[30] Hodo Y, Honda M, Tanaka A, Nomura Y, Arai K, Yamashita T, Sakai Y, Yamashita T, Mizukoshi E, Sakai A, Sasaki M, Nakanuma Y, Moriyama M (2013). Association of interleukin-28B genotype and hepatocellular carcinoma recurrence in patients with chronic hepatitis C. Clin Cancer Res.

[31] Ameres SL, Martinez J, Schroeder R (2007). Molecular basis for target RNA recognition and cleavage by human RISC. Cell.

[32] Figliuzzi M, Marinari E, De Martino A (2013). MicroRNAs as a selective channel of communication between competing RNAs: a steady-state theory. Biophys J.

[33] Meister G, Landthaler M, Patkaniowska A, Dorsett Y, Teng G, Tuschl T (2004). Human Argonaute2 mediates RNA cleavage targeted by miRNAs and siRNAs. Mol Cell.

[34] Zoller M (2009). Tetraspanins: push and pull in suppressing and promoting metastasis. Nat Rev Cancer.

[35] Berditchevski F (2001). Complexes of tetraspanins with integrins: more than meets the eye. J Cell Sci.

[36] Kumari S, Devi Gt, Badana A, Dasari VR, Malla RR (2015). CD151-A Striking Marker for Cancer Therapy. Biomark Cancer.

[37] Yue S, Mu W, Zoller M (2013). Tspan8 and CD151 promote metastasis by distinct mechanisms. Eur J Cancer.

[38] Li Q, Yang XH, Xu F, Sharma C, Wang HX, Knoblich K, Rabinovitz I, Granter SR, Hemler ME (2013). Tetraspanin CD151 plays a key role in skin squamous cell carcinoma. Oncogene.

[39] Zijlstra A, Lewis J, Degryse B, Stuhlmann H, Quigley JP (2008). The inhibition of tumor cell intravasation and subsequent metastasis via regulation of *in vivo* tumor cell motility by the tetraspanin CD151. Cancer Cell.

[40] Sugiura T, Berditchevski F (1999). Function of alpha3beta1-tetraspanin protein complexes in tumor cell invasion. Evidence for the role of the complexes in production of matrix metalloproteinase 2 (MMP-2). J Cell Biol.

[41] Berditchevski F, Odintsova E, Sawada S, Gilbert E (2002). Expression of the palmitoylation-deficient CD151 weakens the association of alpha 3 beta 1 integrin with the tetraspanin-enriched microdomains and affects integrin-dependent signaling. J Biol Chem.

[42] Sarver AL, Subramanian S (2012). Competing endogenous RNA database. Bioinformation.

[43] Agarwal V, Bell GW, Nam JW, Bartel DP (2015). Predicting effective microRNA target sites in mammalian mRNAs. Elife.

[44] Chen C, Ridzon DA, Broomer AJ, Zhou Z, Lee DH, Nguyen JT, Barbisin M, Xu NL, Mahuvakar VR, Andersen MR, Lao KQ, Livak KJ, Guegler KJ (2005). Real-time quantification of microRNAs by stem-loop RT-PCR. Nucleic Acids Res.

