# Supplementary material for: PIK3C2A mRNA functions as a miR-124 sponge to facilitate CD151 expression and enhance malignancy of hepatocellular carcinoma cells
Source: Oncotarget. 2016 May 30;7(28):43376–89. doi: 10.18632/oncotarget.9716 (PMC5190030; doi:10.18632/oncotarget.9716)
Supplement: Supplementary file 1 [file oncotarget-07-43376-s001.pdf]

PIK3C2A mRNA functions as a miR-124 sponge to facilitate CD151 expression and enhance malignancy of hepatocellular carcinoma cells

Supplementary Materials

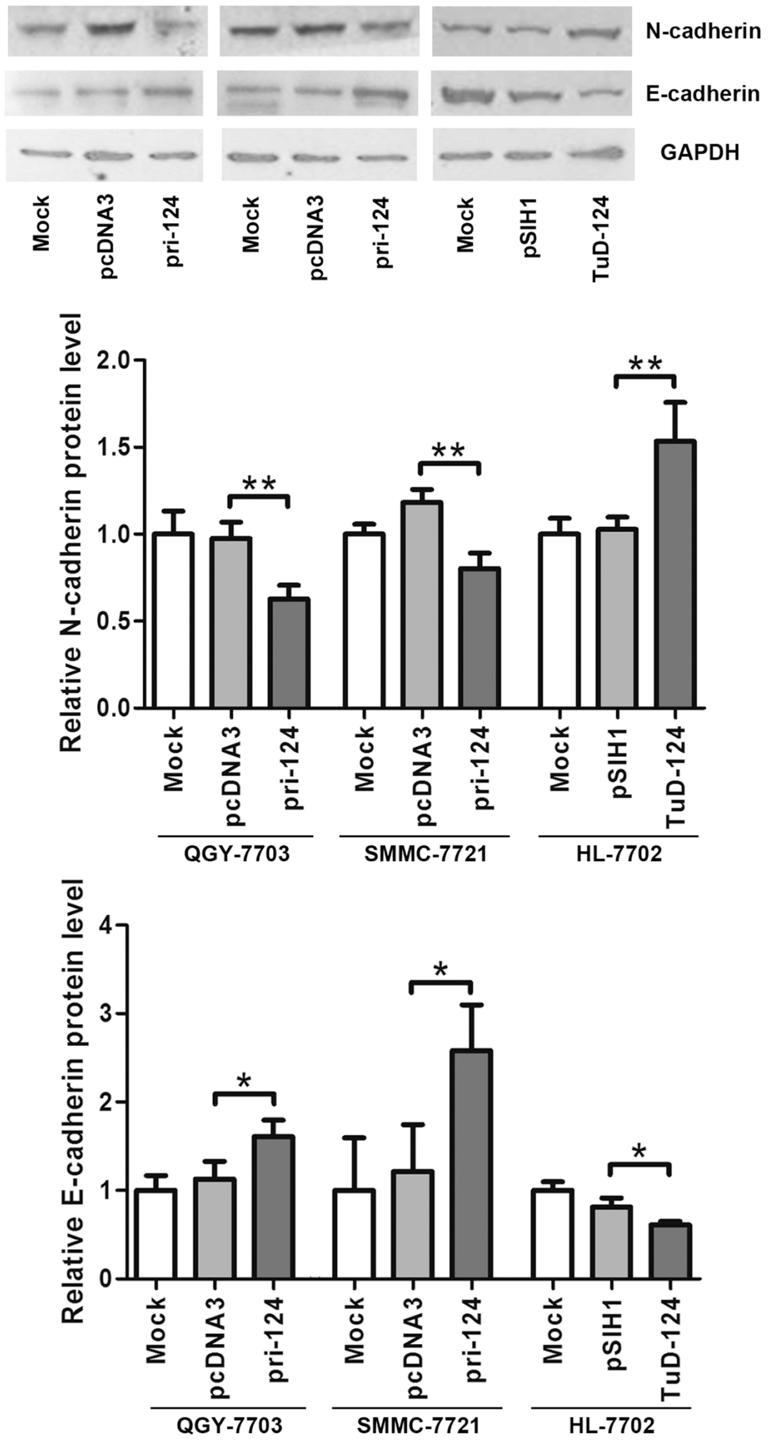

Supplementary Figure S1: Protein level of two epthelial-mesenchymal transition (EMT) markers, N-cadherin and E-cadherin, was detected by Western blot assay. (*$p < 0.05$, **$p < 0.01$).

A

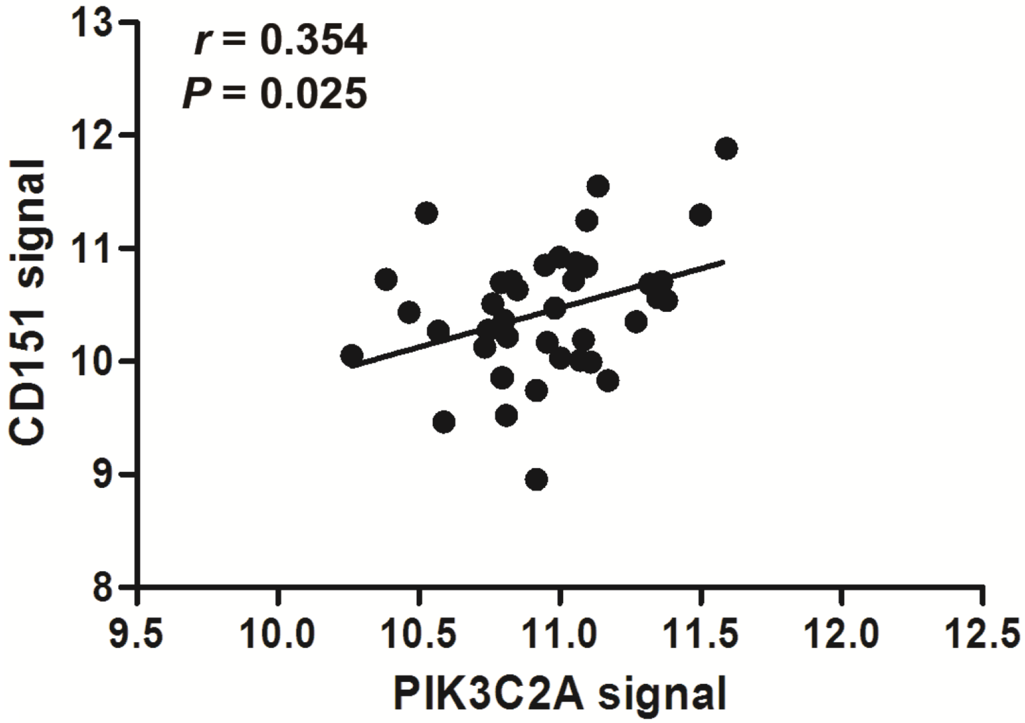

B

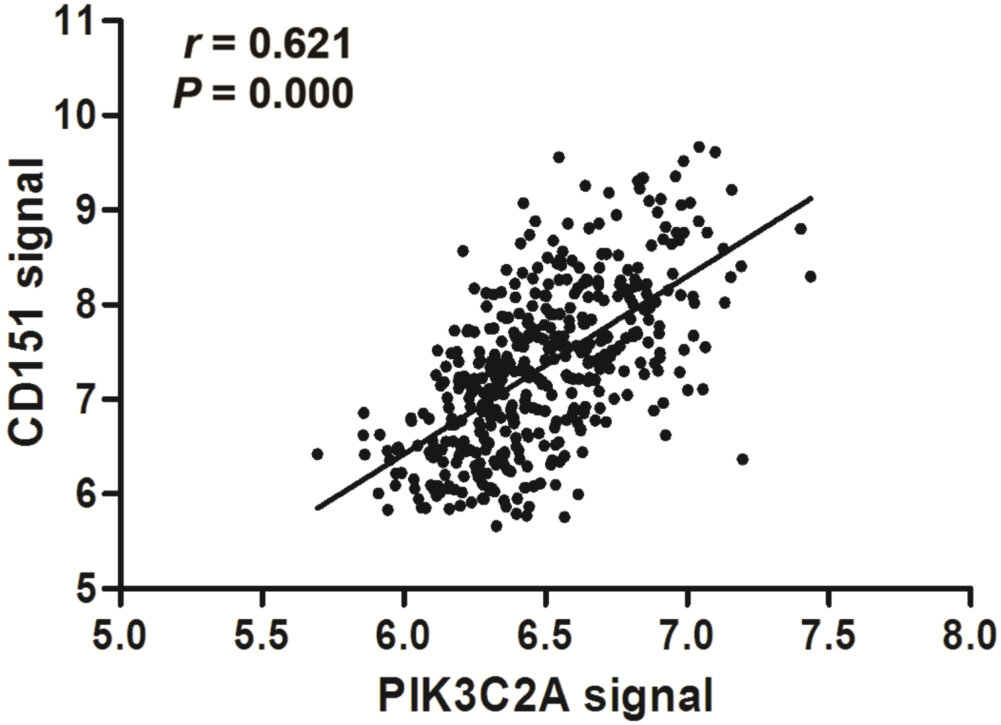

Supplementary Figure S2: Linear correlation analysis between PIK3C2A and CD151 expression levels according to GEO datasets GDS4887 (A) and GSE36376 (B).

Supplementary Table S1: CD151 and PIK3C2A levels in the GEO datasets GDS4887 and GSE36376.

Supplementary Table S2: Sequence of primers and oligonucleotides used in this study

Name	Sequence (5′ to 3′)
CD151-MRE3x-top	GATCCAGCTTCAAGTGCCTTTAGGCAGCTTCAAGTGCCTTTAGGCAGCTTCAAGT GCCTTTAAGCTTG
CD151-MRE3x-bottom	AATTCAAGCTTAAAGGCACTTGAAGCTGCCTAAAGGCACTTGAAGCTGCCTAAA GGCACTTGAAGCTG
pri-124-S	CGAGGATCCAGAGTGGTAATCGCAGTGG
pri-124-A	CGGAATTCCTTTCTGACCCTGAGATGC
TuD-124	GGATCCGACGGCGCTAGGATCATCAACGGCATTCACCGATCGCGTGCCTTACAA GTATTCTGGTCACAGAATACAACGGCATTCACCGATCGCGTGCCTTACAAGA TGATCCT AGCGCCGTCTTTTTGAATTC
EGFP-CDS-S	GCAGCCAAGCTTGCCACCATGTGTAGCAAGGGC
EGFP-CDS-A	CGCGGATCCTTTACTTGTACAGCTCGTCC
PIK3C2A-MRE1wt-top	GATCCTGATGTTGTAAATAATGGTGCCTTAACTG
PIK3C2A-MRE1wt-bottom	AATTCAGTTAAGGCACCATTATTTACAACATCAG
PIK3C2A-MRE2wt-top	AATTCTTAATTTAACTCATTTTGTGCCTTCTTTC
PIK3C2A-MRE2wt-bottom	TCGAGAAAGAAGGCACAAAATGAGTTAAATTAAG
PIK3C2A-MRE3wt-top	TCGAGATAAAAGTTTCAATATCTTGCCTTAAATT
PIK3C2A-MRE3wt-bottom	CTAGAATTTAAGGCAAGATATTGAAACTTTTATC
PIK3C2A-MRE1/2mut-top	GATCCTGATGTTGTAAATAATGCAGGGATAACTGAATTC TTAATTTAACTCATTTT CAGGGATCTTTC
PIK3C2A-MRE1/2mut-bottom	TCGAGAAAGATCCCTGAAAATGAGTTAAATTAAGAAT TCAGTTATCCCTGCATTAT TTACAACATCAG
PIK3C2A-MRE2/3mut-top	AATTCTTAATTTAACTCATTTTCAGGGATCTTTCTCGAG ATAAAAGTTTCAATATCT AGGGATTAATT
PIK3C2A-MRE2/3mut-bottom	CTAGAATTAATCCCTAGATATTGAAACTTTTATCTCGAG AAAGATCCCTGAAAAT GAGTTAAATTAAG
PIK3C2A-MRE1mut-top	GATCCTGATGTTGTAAATAATGCAGGGATAACTG
PIK3C2A-MRE1mut-bottom	AATTCAGTTATCCCTGCATTATTTACAACATCAG
PIK3C2A-MRE3mut-top	TCGAGATAAAAGTTTCAATATCTAGGGATTAATT
PIK3C2A-MRE3mut-bottom	CTAGAATTAATCCCTAGATATTGAAACTTTTATC
CD151-MREwt-top	GATCCAGCTTCAAGTGCCTTTAAGCTTG
CD151-MREwt-bottom	AATTCAAGCTTAAAGGCACTTGAAGCTG
CD151-MREmut-top	GATCCAGCTTCAACAGGGATTAAGCTTG
CD151-MREmut-bottom	AATTCAAGCTTAATCCCTGTTGAAGCTG
shR-PIK3C2A-top	GATCCGCAGGATTATGATCTCATGTTCAAGAGACATGAGATCATAATCCTGCTTTT TTGAATTCA
shR-PIK3C2A-bottom	AGCTTGAATTCAAAAAAGCAGGATTATGATCTCATGTCTCTTGAACATGAGATCAT AATCCTGCG
shR-CD151-top	GATCCGAAGACAACATGTGGCACCTTCAAGAGAGGTGCCACATGTTGTCTTCTTT TTTGGATCCA
shR-CD151-bottom	AGCTTGGATCCAAAAAAGAAGACAACATGTGGCACCTCTCTTGAAGGTGCCACAT GTTGTCTTCG
shR-NC-top	GATCCGTTCTCCGAACGTGTCACGTTTCAAGAGAACGTGACACGTTCGGAGAATTT TTTGGATCCA
shR-NC-bottom	AGCTTGGATCCAAAAAATTCTCCGAACGTGTCACGTTCTCTTGAAACGTGACACGT TCGGAGAACG
CD151-CDS-S	ACTGAATTCGCCACGATGGGTGAGTTCAACGAG
CD151-CDS-A	CAGTCTAGATCAGTAGTGCTCCAGCTTGAGACTC
miR-124-RT	GTCGTATCCAGTGCAGGGTCCGAGGTGCACTGGATACGACGGCATTC
U6-RT	GTCGTATCCAGTGCAGGGTCCGAGGTGCACTGGATACGACAAAATATGG

miR-124-Fwd	TGCGGTAAGGCACGCGGTG U6-Fwd TGCGGGTGCTCGCTTCGGCAGC
Reverse	CCAGTGCAGGGTCCGAGGT
CD151-qPCR-S	TCGGCATTGCCTGTGTGC
CD151-qPCR-A	ACAGGGTGGGTGTCATCAGG
CTDSP1-qPCR-S	GTGCCAACCCATCTCCTAC
CTDSP1-qPCR-A	GACATCACCACTTGACATCC
PIK3C2A-qPCR-S	TGAGGAACCCAATGTAGAAC
PIK3C2A-qPCR-A	GGCAACTAATTTAAGGCAAG
PTPN12-qPCR-S	TCAAAGTCAGGAACGATCTG
PTPN12-qPCR-A	CCTTTGGGTTTTCCACATC
OSBPL3-qPCR-S	TGCGTCATGTAAATATCTG
OSBPL3-qPCR-A	ACATTCAACTAGCTACATGAG
CEBPA-qPCR-S	ACACGAAGCACGATCAGTC
CEBPA-qPCR-A	TCATTTTGGCAAGTATCCG
LAMC1-qPCR-S	CATGTATCTGAATACCTCC
LAMC1-qPCR-A	CATAGCTTGTCAACCTG
CBL-qPCR-S	TTCTAAGGCAGCACTGTATC
CBL-qPCR-A	ACACTGCACCTGCATGTC
NFATC1-qPCR-S	AGTAGCTGTAGGTTACCAG
NFATC1-qPCR-A	TCATCTGAAAACACACAAC
NR3C1-qPCR-S	TACAAGCAGAACTGAGGCAC
NR3C1-qPCR-A	AATTGCTCCCTGCCTCTG
SNAI2-qPCR-S	AGAATGCATTTCTTCACTC
SNAI2-qPCR-A	CTCAATCTAGCCATCAGC
SOS1-qPCR-S	GACTACGCAAGTATAGGTC
SOS1-qPCR-A	TGCTTCTTAAGTAGAGCTC
CD151-MRE3x-qPCR-S	GATCCAGCTTCAAGTG
CD151-MRE3x-qPCR-A	CAAGCTTAAAGGCAC

S: sense primer; A: antisense primer.
